# ASFV Gene A151R Is Involved in the Process of Virulence in Domestic Swine

**DOI:** 10.3390/v14081834

**Published:** 2022-08-21

**Authors:** Elizabeth Ramirez-Medina, Elizabeth Vuono, Sarah Pruitt, Ayushi Rai, Nallely Espinoza, Alyssa Valladares, Edward Spinard, Ediane Silva, Lauro Velazquez-Salinas, Douglas P. Gladue, Manuel V. Borca

**Affiliations:** 1 Plum Island Animal Disease Center, ARS, USDA, Greenport, NY 11944, USA; 2 Department of Pathobiology and Population Medicine, Mississippi State University, P.O. Box 6100, Starkville, MS 39762, USA; 3 Oak Ridge Institute for Science and Education (ORISE), Oak Ridge, TN 37830, USA

**Keywords:** ASFV, ASF, African swine fever virus, A151R, helicase

## Abstract

African swine fever virus (ASFV) is the etiological agent of a swine pandemic affecting a large geographical area extending from Central Europe to Asia. The viral disease was also recently identified in the Dominican Republic and Haiti. ASFV is a structurally complex virus with a large dsDNA genome that encodes for more than 150 genes. Most of these genes have not been experimentally characterized. One of these genes, A151R, encodes for a nonstructural protein and has been reported to be required for the replication of a Vero-cell-adapted ASFV strain. Here, we evaluated the role of the A151R gene in the context of the highly virulent field isolate Georgia 2010 (ASFV-G) during virus replication in swine macrophage cell cultures and during experimental infection in swine. We show that the recombinant virus ASFV-G-∆A151R, harboring a deletion of the A151R gene, replicated in swine macrophage cultures as efficiently as the parental virus ASFV-G, indicating that the A151R gene is not required for ASFV replication in swine macrophages. Interestingly, experimental infection of domestic pigs demonstrated that ASFV-G-∆A151R had a decreased replication rate and produced a drastic reduction in virus virulence. Animals were intramuscularly inoculated with 10^2^ HAD_50_ of ASFV-G-∆A151R and compared with pigs receiving a similar dose of virulent ASFV-G. All ASFV-G-infected pigs developed an acute lethal form of the disease, while those inoculated with ASFV-G-∆A151R remained healthy during the 28-day observational period, with the exception of only one showing a protracted, but fatal, form of the disease. All ASFV-G-∆A151R surviving animals presented protracted viremias with lower virus titers than those detected in ASFV-G-infected animals. In addition, three out of the four animals surviving the infection with ASFV-G-∆A151R were protected against the challenge with the virulent parental virus ASFV-G. This is the first report indicating that the ASFV A151R gene is involved in virus virulence in domestic swine, suggesting that its deletion may be used to increase the safety profile of currently experimental vaccines.

## 1. Introduction

African swine fever virus (ASFV) is producing a devastating pandemic affecting the swine industry in a large geographical area from Central Europe to East and Southeast Asia. ASFV was recently detected in the Dominican Republic and Haiti, the first outbreak of ASF in America in the last 40 years [1]. With a commercial vaccine available in only Vietnam [2,3], disease control in general depends on culling susceptible animals and strict biosecurity procedures to avoid disease spread. 

ASFV is a large and structurally complex dsDNA virus (approximately 180 kb) [4] that encodes for more than 150 genes, most of them yet to be characterized [1,4]. Production of recombinant ASFV harboring gene deletion(s) has been a powerful method to understand the role of specific genes in virus replication and virulence during natural infection. This approach has been used for the rational development of multiple ASFV experimental vaccines [5]. The resulting attenuated viruses have been shown to be effective in preventing disease during challenges with parental virulent strains [6,7,8,9,10,11,12]. Elucidating the role of virus genes involved in virus replication and/or virulence has allowed for the development of experimental vaccines [6,7,8,9,10,11,12] and expanded the overall knowledge of virus function [13,14,15,16,17,18,19,20,21,22,23,24,25].

ASFV gene A151R encodes for a non-structural protein that has been associated with virus replication [26]. Inhibition of A151R translation by siRNA significantly decreased virus production in cell cultures. These results were obtained using an ASFV strain (BA71V) adapted to grow in the Vero cell line, suggesting an important role of this gene in virus replication [27]. Although its crystal structure has been recently reported, the protein encoded by the A151R gene shares a very low sequence identity with homologous structures [26]. In this report, we investigated the role of the A151R gene in ASFV replication in swine macrophage cultures, the natural target cells for the virus, and in experimental infection of domestic pigs. The studies presented here demonstrate that the ASFV gene A151R is involved in both virus replication and disease production in domestic swine.

## 2. Materials and Methods

### 2.1. Viruses and Cells

Production of primary blood-derived swine macrophage cultures was done as described elsewhere [28]. Briefly, the peripheral blood mononuclear cell fraction was enriched using Ficoll-Paque (Pharmacia, Piscataway, NJ, USA) gradients, and adherent cells were obtained by overnight incubation in Primaria T75 flasks. Adherent cells were detached by EDTA-PBS treatment and seeded into Primaria T25 6- or 96-well dishes at a density of 5 × 10^6^ cells per mL. ASFV Georgia (ASFV-G) was a field isolate kindly provided by Dr. Nino Vepkhvadze, from the Laboratory of the Ministry of Agriculture in Tbilisi, Republic of Georgia [29]. Comparative growth curves between recombinant ASFV-G-∆A151R and parental ASFV-G were assessed in primary swine macrophage cultures in 24-well Primaria plates using an MOI of 0.01 HAD_50_ (hemadsorbing doses, as determined in primary swine macrophage cell cultures). After 1 h of adsorption at 37 °C under 5% CO_2_, the inoculum was removed, cells rinsed twice with PBS, and incubated with macrophage media at 37 °C under 5% CO_2_. At different times post-infection, the cells were frozen at ≤−70 °C, thawed, and the lysates titrated by HAD_50_/mL in primary swine macrophage cell cultures in 96-well plates. All samples were run simultaneously to avoid inter-assay variability. The presence of the virus was assessed by hemadsorption (HA), and virus titers were calculated as previously described [30].

### 2.2. Detection of A151R Transcription 

Real-time PCR (qPCR) was used to assess the transcriptional profile of the A151R gene in cultures of porcine macrophage cell cultures infected with ASFV-G. As a control for transcription, the early CP204L (p30) and late B646L (p72) genes were also quantified. Cell cultures of porcine macrophages were infected (MOI of 1) with ASFV-G. RNA extractions, using an RNeasy Kit (QIAGEN, Hilden, Germany), were performed at 4-, 6-, 8-, and 24 h post-infection. All extracted materials were treated with 2 units of DNase I (BioLabs, Ipswich, MA, USA) and then purified using the Monarch^®^ RNA Cleanup Kit (New England BioLabs, Inc.). One μg of RNA was used to produce cDNA using qScript cDNA SuperMix (Quanta bio, Beverly, MA, USA), which was used for the qPCR. 

Primers and probe for detection of the A151R gene were designed using the ASFV Georgia 2007/1 strain (GenBank data base NC_044959.2). Primer forward: 5′-GAGCCGCGTACTCAAATTTATT-3′, reverse: 5′-AGTACACCAAATGCTACGATCAT-3′, and probe: 5′-FAM/TGGAATGTTTCAACTTCAGTCGGTCCTC/MGBNFQ-3′. Primers and probes for the detection of p72, p30, and the β-actin gene were previously described [12]. All qPCRs were conducted using the TaqMan Universal PCR Master Mix (Applied Biosystems) using the following amplification conditions: one step at 55 °C for 2 min, followed by one denaturation step at 95 °C for 10 min, then 40 cycles of denaturation at 95 °C for 15 s and annealing/extension at 65 °C for 1 min.

### 2.3. Construction of the ASFV A151R Deletion Mutant 

An ASFV strain lacking the A151R gene, ASFV-G-∆A151R, was developed by homologous recombination between the genome of the parental ASFV-G and a recombination transfer vector [9]. The recombinant transfer vector, p72mCherryΔ151R, contains the flanking genomic regions of the A151R gene with the left arm covering genomic positions 48629-49629 and the right arm between genomic positions 50108-51108, and contains a reporter gene cassette harboring the mCherry fluorescent protein (mCherry) gene under the control of the ASFV p72 late gene promoter [31]. The recombinant transfer vector was obtained by DNA synthesis (Epoch Life Sciences, Sugar Land, TX, USA). The designed construction creates a deletion of 478 nucleotides between nucleotide positions 49630 and 50107, deleting the A151R ORF sequence. ASFV-G-∆A151R was purified to homogeneity by successive rounds of limiting dilution based on detection of the mCherry activity. ASFV DNA was full-length-sequenced using next-generation sequencing (NGS) as previously described [31] using an Illumina NextSeq500 sequencer. Sequence analysis was performed using CLC Genomics Workbench software version 20 (QIAGEN, Hilden, Germany). 

### 2.4. Animal Experiments

The virulence of the recombinant ASFV-G-∆A151R was assessed in 35–40 kg commercial breed swine. Five pigs were intramuscularly (IM) inoculated with 10^2^ HAD_50_ of ASFV-G-∆A151R while another group of five animals was inoculated with 10^2^ HAD_50_ of ASFV-G. Development of ASF clinical signs (anorexia, depression, fever, purple skin discoloration, staggering gait, diarrhea, and cough) as well as changes in body temperature were recorded daily throughout the experiment. Blood samples were obtained at 0, 4, 7, 11, 14, 21, and 28 days post-inoculation (pi). In protection experiments, animals inoculated with ASFV-G-∆A151R were IM challenged 28 days later with 10^2^ HAD_50_ of parental virulent ASFV-G. The presence of clinical signs associated with the disease was recorded as described earlier. Animal experiments were performed under biosafety level 3 conditions in the animal facilities at Plum Island Animal Disease Center, following a strict protocol approved by the Institutional Animal Care and Use Committee (225.01-16-R_090716).

## 3. Results and Discussion

### 3.1. A151R Gene Is Conserved across Different ASFV Isolates

**Figure 1:**
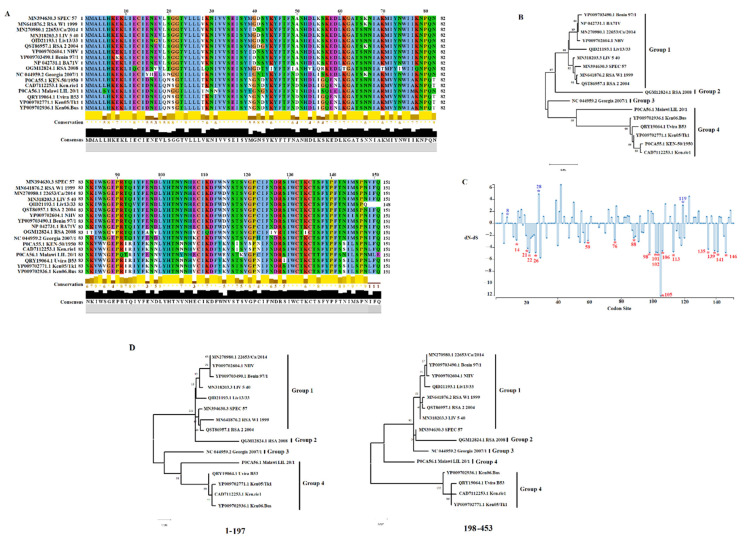
Evaluation of A151R protein across ASFV isolates. (**A**) Amino acid alignment representing the diversity of A151R protein of ASFV in the field. Residues in white spots represent changes between amino acids with different charges. Conservation plot scores reflect the nature of the change in specific sites, with high scores associated with changes in similar biological properties. Alignment was produced using the software Jalview version 2.11.1.4. (**B**) Phylogenetic analysis representing the diversity of A151R protein of ASFV in the field. Based on the cluster distribution, isolates were categorized into five groups. Numbers above internal branches represent bootstrap values (1000 repetitions). (**C**) The graphic represents the dN (rate of evolution at non-synonymous sites), dS (rate of evolution at synonymous sites), and ratio (dN-dS) at specific codon sites in the A151R gene of ASFV. Blue and red asterisks represent codon sites evolving under diversifying and purifying selection, respectively. Analyses were conducted using the evolutionary algorithms FEL and MEME using cutoff values of *p* = 0.1. (**D**) Phylogenetic analysis showing the topology incongruence produced by different segments where the single breakpoint at nucleotide 197 was detected by GARD. Phylogenetic analysis was conducted with the maximum likelihood method, using the general time reversible model.

Previously, the A151R protein was identified as a non-structural protein, expressed during early and late stages of viral replication, with a potential role in replication and virus assembly [26]. To assess the conservation of the A151R gene across different ASFV isolates, 17 ASFV isolates representing the genetic diversity of this gene were evaluated by pairwise analysis using the model p-distance and the bootstrap method to obtain a confidence interval of 95% [32]. We calculated an overall 80.79–99.77% (average 90.88%), and 65.56–99.33% (average 84.70%) nucleotide and amino acid identity, respectively (Figure 1A), across the 17 isolates analyzed. When evaluated by protein BLAST analysis, A151R was 100% identical among 41 contemporary ASFV isolates associated with the pandemic Eurasia lineage (genotype II), indicating high conservation of this gene within this lineage. The China/GD/2019 isolate was the only exception, with 98.90% nucleotide identity when compared with the other 41 isolates. This difference is due to a five-nucleotide deletion (nucleotides 270 to 274) that truncates the protein. In addition, deletion in this gene has been reported in isolates (such as ASFV Liv/13/33) of other ASFV genotypes (Figure 1A).

Based on a phylogenetic analysis conducted by maximum likelihood and the JTT model using the amino acid sequences of A151R isolates, we were able to categorize multiple isolates into four different groups (Figure 1B). Interestingly, the Georgia 2007/1 isolate, a virus of the genotype II lineage, is a unique isolate classified into Group 3, highlighting the differences between the A151R protein of the pandemic lineage compared to other ASFV isolates. This is a significant observation, considering that previous studies proposed that A151R is essential for the in vitro replication of the isolate BA71V (Group 1) [27], which contrasts with the results obtained in our study. We consider the low protein homology (90.07%) a potential reason for the disparity. It is important to consider that differences in other parts of each isolate’s genome might also explain the differences. 

Little is known about the function and structural mechanism of the A151R protein in the replication of ASFV. Just recently, a study identified the motif WCTKC at the C’ terminus of this protein (amino acids 131–135), which is similar to the thioredoxins’ active site motif [26]. Our attempts to find a homology with other proteins using the program Pfam [33] were negative when A151R was compared with 19,175 other protein families. To gain more insight into the A151R protein, we conducted an evolutionary analysis. The fixed-effects likelihood (FEL) and mixed-effects model of evolution (MEME) [34,35] algorithms indicated that the evolution of the A151R gene is influenced by negative selection (dN/dS = 0.601), suggesting an increased number of synonymous mutations were fixed during the evolution of this gene and favored the preservation of the A151R protein. Our analyses identified 17 codons evolving under purifying selection (Figure 1C); these sites may represent relevant sites for the functionality of the A151R protein preserved by the evolutionary process. We identified amino acid 135 evolving under purifying selection, preserving the conservation of the WCTKC motif. However, a mutation present in the RSA 2008 isolate changed the sequence of the motif WCTKC to WCTIC. More studies are necessary to understand the impact of this mutation. Furthermore, amino acids were identified that are evolving under diversifying selection, potentially promoting the divergence of this protein. Interestingly, using the genetic algorithm for recombination detection (GARD) [36], we identified evidence of recombination. A single breakpoint was identified by GARD at nucleotide 197. This inference was supported by an improvement in the c-AIC = 3030.73 assuming the existence of potential breakpoints vs. AIC-c = 3046.09 model assuming a single partition: no breakpoints. This inference is consistent with the topology incongruence produced by different segments at the A151R gene (Figure 1D). This result indicates the potential relevance of recombination in the evolution of this gene.

### 3.2. Detection of A151L Transcription

**Figure 2:**
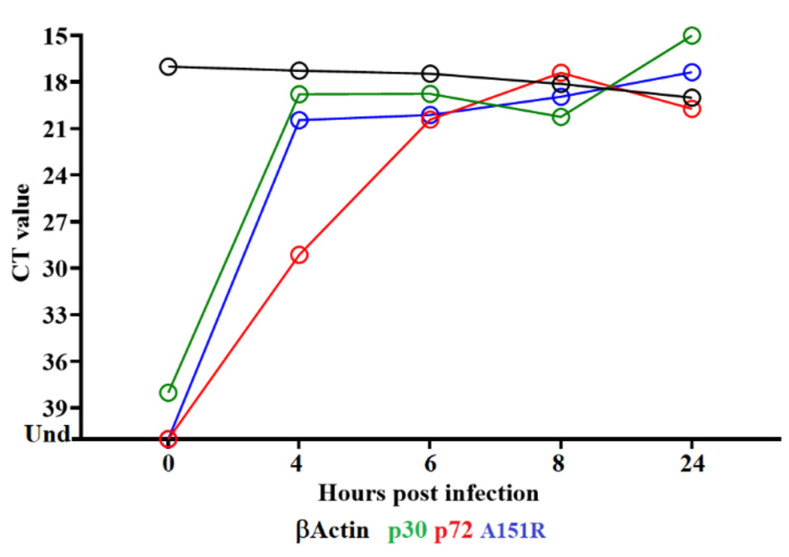
Expression profile of A151R gene of ASFV during in vitro infection of porcine macrophages. Reverse transcription followed by qPCR was used to evaluate the expression profile of the A151R gene during in vitro infection at different time points, up to 24 h. As a reference for this analysis, we used qPCRs to specifically detect the expression of genes encoding ASFV proteins p30 (early expression) and p72 (late expression). Additionally, the β-actin gene was used as a control to evaluate the quality and levels of RNA during the infection at different time points.

To determine when the A151R gene is transcribed during the replication cycle, a time course experiment was performed to analyze the kinetics of RNA transcription in primary swine macrophages infected with the ASFV strain Georgia. Swine macrophage cultures were infected at an MOI = 1 with ASFV-G, and cell lysate samples were taken at 0, 4, 6, 8, and 24 hpi. The presence of A151R RNA was detected by two-step RT-PCR as described in the Material and Methods Section. Transcription of A151R was detected at 4 hpi and remained stable until 24 hpi (Figure 2). The pattern of expression of the well-characterized ASFV early protein p30 (CP204L) and the late protein p72 (B646L) has been previously described and is used here as a reference of early and late transcription profiles, respectively. Expression of A151R was transiently detected throughout the assessed timepoints, suggesting that it is neither a late nor an early protein. As previously reported [26], our results indicate that the ASFV A151R gene encodes for a protein that is expressed throughout the virus replication cycle.

### 3.3. Development of the ASFV-G-ΔA151R Deletion Mutant

Conservation of the A151R gene among ASFV isolates and its previously described involvement in the process of virus replication [26,27] support the hypothesis that A151R could play a role in several virus functions.

**Figure 3:**
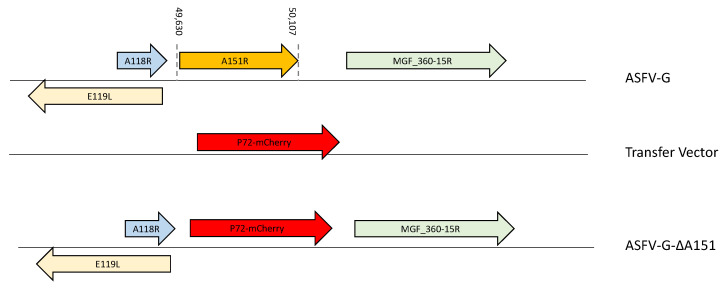
Schematic for the development of ASFV-G-∆A151R. The transfer vector contains the p72 promoter and a mCherry cassette; the flanking left and right arms are indicated and were designed to have flanking ends at both sides of the deletion/insertion cassette. The nucleotide positions of the ASFV-G genome are indicated. The resulting ASFV-G-∆A151R virus with the cassette inserted is shown on the bottom.

A recombinant deletion mutant of the highly virulent ASFV Georgia 2007 isolate (ASFV-G) lacking the A151R gene was developed (ASFV-G-∆A151R). The A151R gene was deleted by substituting 151 amino acid residues in the A151R ORF with a p72mCherry cassette by homologous recombination and 23 nucleotides of the immediate promoter region for A151 (Figure 3). As a result, a 478bp DNA fragment (found between nucleotide positions 49,630 and 50,107 and deleted from the ASFV-G genome, resulting in the deletion of the entire A151R ORF. This deleted fragment was replaced by a 1226-bp cassette containing the p72mCherry construct (see Section 2) (Figure 3). The recombinant ASFV-G-∆A151R stock was obtained after several limiting dilution steps in primary swine macrophage cell cultures. Final virus stock was also produced in primary swine macrophage cell cultures.

The accuracy of the genomic alterations introduced into ASFV-G-∆A151R and the integrity of the remaining virus genome were evaluated by analyzing the full virus genome sequence by NGS using an Illumina NextSeq^®^ 500. Analysis of the ASFV-G-∆A151R genome confirmed a deletion of 478 nucleotides, the designed genomic modifications. In addition, the genome of ASFV-G-∆A151R had an insertion of 1226 nucleotides, consistent with the p72-mCherry cassette sequence. No other genomic differences were detected between ASFV-G-∆A151R and ASFV-G, confirming no unwanted genomic changes were introduced in the development and further purification of ASFV-G-∆A151R. NGS analysis also showed the absence of the parental ASFV-G genome as a possible contaminant in the ASFV-G-∆A151R stock.

### 3.4. Replication of ASFV-G-∆A151R in Primary Swine Macrophages

**Figure 4:**
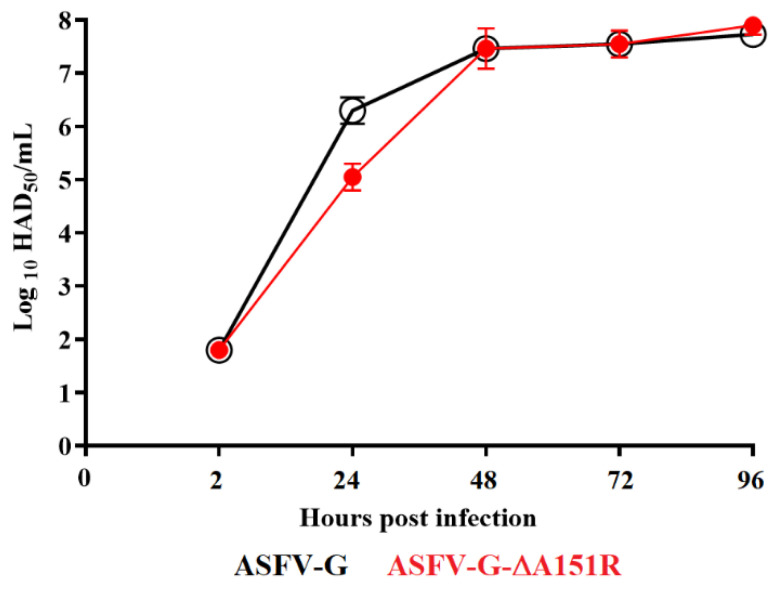
In vitro growth kinetics in primary swine macrophage cell cultures for ASFV-G-∆A151R and parental ASFV-G (MOI = 0.01). Samples were taken from three independent experiments at the indicated time points and titrated. Data represent means and standard deviations of three replicas. Sensitivity using this methodology for detecting virus was ≥log10 1.8 HAD_50_/mL. No significant differences in viral yields between viruses were observed at any time point tested, which was determined using the Holm–Sidak method (α = 0.05) without assuming a consistent standard deviation. All calculations were conducted using the software Graphpad Prism version 8.

To assess the potential role of A151R in the process of ASFV replication, the kinetics of ASFV-G-∆A151R replication was compared with that of the parental ASFV-G. A multistep growth curve in swine macrophage cultures was performed. Cell cultures were infected with an MOI of 0.01, either with the recombinant ASFV-G-∆A151R or the parental ASFV-G. Virus yields were assessed at 2, 24, 48, 72, and 96 h post-infection (pi). ASFV-G-∆A151R had a growth kinetic indistinguishable from that of the parental ASFV-G, with no significant differences in virus titers at any of the evaluated times post-infection (Figure 4). Results demonstrate that deletion of the A151R gene from the ASFV-G genome does not affect the ability of ASFV-G-∆A151R to replicate in primary cultures of swine macrophages. This was unexpected, since it had previously been reported that the A151R gene is involved in ASFV replication [26,27]. Of note, those results were obtained by blocking A151R transcription using siRNA targeting and an ASFV strain (BA71V) adapted to growth in an established Vero cell line. Adaptation of the virulent BA71 strain to replication in Vero cells resulted in dramatic genomic changes and attenuation of the virus in domestic swine [37]. It is possible the critical role of A151R in BA71V replication in Vero cells is associated with loss of genes potentially replacing A151R function. 

### 3.5. Assessment of ASFV-G-∆A151R Virulence in Swine

**Figure 5:**
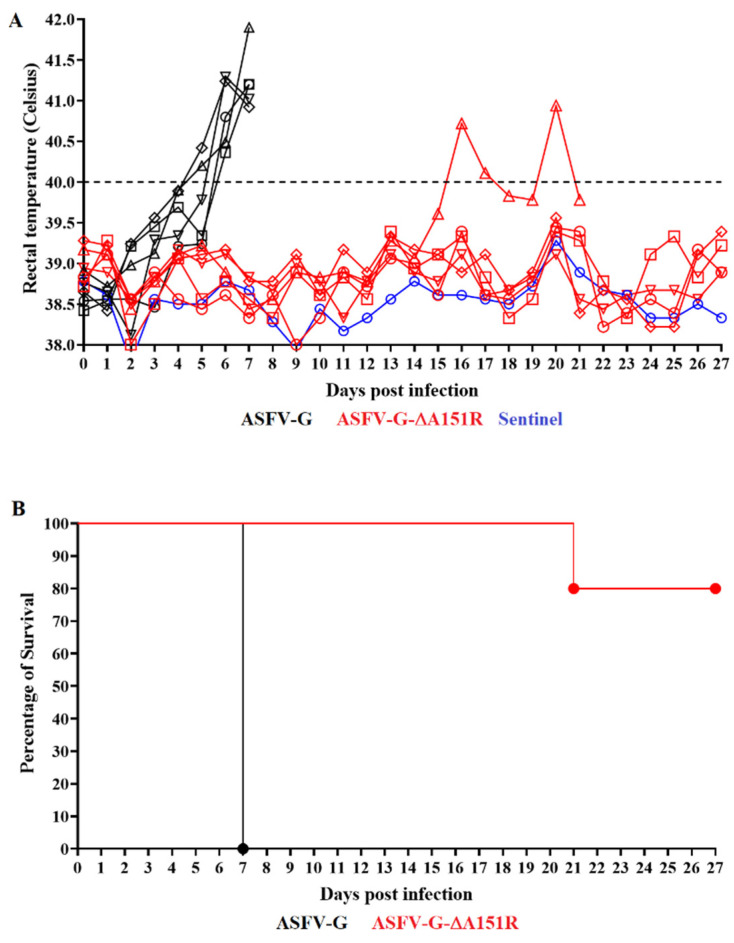
Evolution of body temperature (**A**) and mortality (**B**) in animals (5 animals/group) IM infected with 10^2^ HAD_50_ of either ASFV-G-∆A151R or parental ASFV-G.

To evaluate the impact of deletion of the A151R gene from the genome of ASFV-G, ASFV-G-∆A151R was inoculated into domestic swine. Five 30–40 kg pigs were IM inoculated with 10^2^ HAD_50_ of either ASFV-G-∆A151R or the parental ASFV-G. All animals inoculated with virulent ASFV-G presented a sudden rise in body temperature (>40 °C) for day 5–6 pi, which was quickly followed by the development of full clinical disease (depression, anorexia, staggering gait, diarrhea, and purple skin discoloration) (Table 1 and Figure 5). Disease severity rapidly progressed to a terminal disease, with all animals euthanized in extremis by day 7 pi.

Animals inoculated with ASFV-G-∆A151R remained clinically normal during the 28-day observational period with the exception of one animal that presented a protracted but lethal form of the disease. This animal presented a rise in body temperature by day 16 pi followed by the development of a severe form of the disease and needed to be euthanized on day 21 pi (Table 1 and Figure 5). These results indicate that deletion of the A151R gene from the genome of ASFV-G causes an extensive decrease in virus virulence in domestic swine.

**Figure 6:**
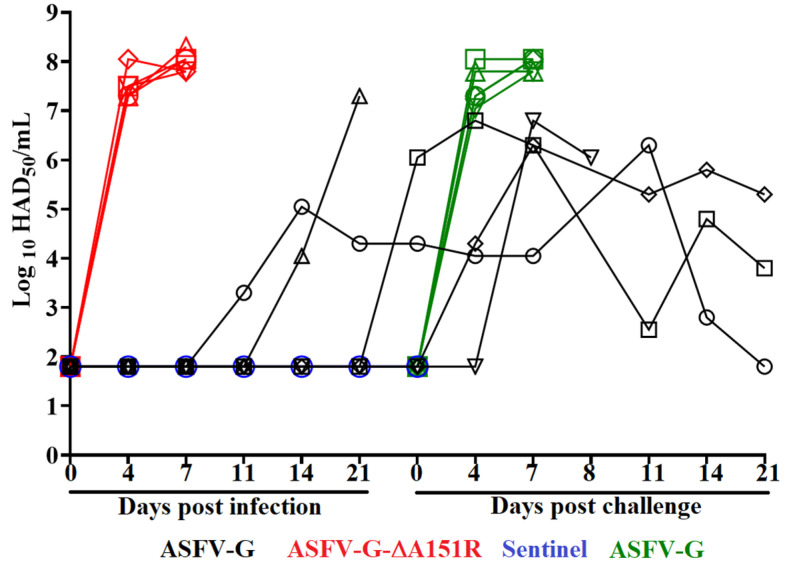
Viremia titers detected in pigs IM inoculated with 10^2^ HAD_50_ of either ASFV-G-∆A151R (black symbols) or ASFV-G (red and green symbols). Each symbol represents the average of animal titers in each of the groups. Sensitivity of virus detection: >log10 1.8 TCID_50_/mL.

Virus replication in animals experimentally infected with either ASFV-G-∆A151R or parental ASFV-G was evaluated by quantifying titers of viremia at different times post-infection. As expected, viremia titers in IM inoculated ASFV-G-infected animals were high (10^7.3^–10^8.3^ HAD_50_/mL) on day 4 pi, and remained high by day 7 pi (around 10^8^ HAD_50_/mL), when all animals were euthanized. In the ASFV-G-∆A151R-inoculated animals, viremia values and kinetics were heterogenous and followed the presentation of the clinical signs of the disease. The animal presented a protracted form of the disease and showed an undetectable level of viremia until day 14 pi (showing a titer of 10^4.05^ HAD_5__0_/mL). Titers increased to 10^7.3^ HAD_50_/mL by day 21 pi, when the animal was euthanized due to the severity of the clinical signs. It is important to mention that NGS analysis of the virus obtained from this animal showed the exclusive presence of ASFV-G-∆A151R, eliminating the possibility that the disease of this particular animal was caused by the presence of residual parental ASFV-G in the ASFV-G-∆A151R stock. It is possible that ASFV-G-ΔA151R retains some degree of residual virulence that may produce a delayed form of the disease in an animal that may present a higher susceptibility than the others. The rest of the ASFV-G-∆A151R-inoculated animals remined clinically asymptomatic. One animal presented viremias titers of 10^5.05^, 10^4.3^, and 10^4.3^ HAD_50_/mL on day 14, 21, and 28 pi, respectively. The third animal presented undetectable viremias until day 28 pi when it reached a titer of 10^6.05^ HAD_50_/mL, while the other two animals did not show any detectable viremia titers during the 28-day observational period (Figure 6). 

**Figure 7:**
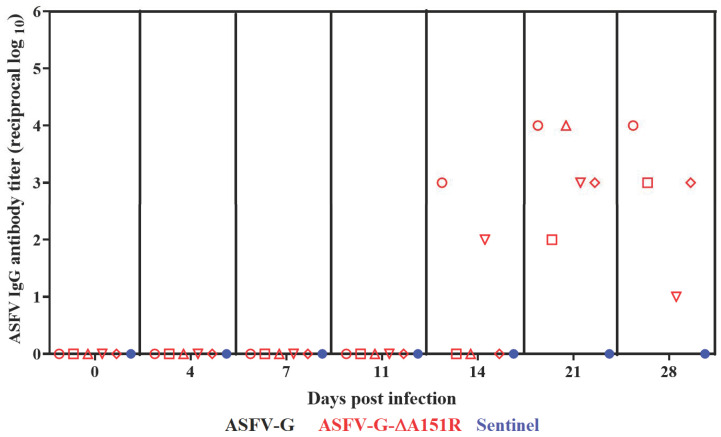
Anti-ASFV antibody titers detected by ELISA in pigs IM inoculated with 10^2^ HAD_50_ of ASFV-G-ΔA151R. Each point represents values from individual animals.

In most of the cases, animals surviving the infection with ASFV presented a strong virus-specific antibody response [7,8,9,12,38,39]. The assessment of ASFV-specific antibodies in serum of the ASFV-G-∆A151R-inoculated pigs was performed using an in-house-developed ELISA [40]. All surviving pigs developed an ASFV-specific circulating antibody response. The response could be detected by day 14 pi in two of these animals. All animals presented strong antibody titers by day 21 pi and remained high until reaching day 28 pi (Figure 7).

It should be noted that a naïve animal (sentinel), which had been cohabitating with the ASFV-G-∆A151R-inoculated pigs since day 0 pi, remained completely asymptomatic, not showing detectable viremic titers nor the presence of circulating ASFV-specific antibodies during the whole observational period of 28 days. This indicates the absence of transmission of the ASFV-G-∆A151R from the inoculated pigs, particularly for the animal that developed clinical disease and needed to be euthanized by day 21 pi. 

### 3.6. Evaluation of the Protective Effect of the Infection with ASFV-G-ΔA151R

In general, pigs surviving an infection with an attenuated ASFV strain became protected against an experimental infection with the virulent parental virus [6,7,8,9,10,11,12]. It was then important to evaluate if animals infected with ASFV-G-ΔA151R were protected against the challenge with the highly virulent ASFV-G. All four pigs surviving infection with ASFV-G-ΔA151R were IM challenged with 10^2^ HAD_50_ of ASFV-G. An additional group of five naïve animals was used as a control group and challenged under the same conditions. 

**Figure 8:**
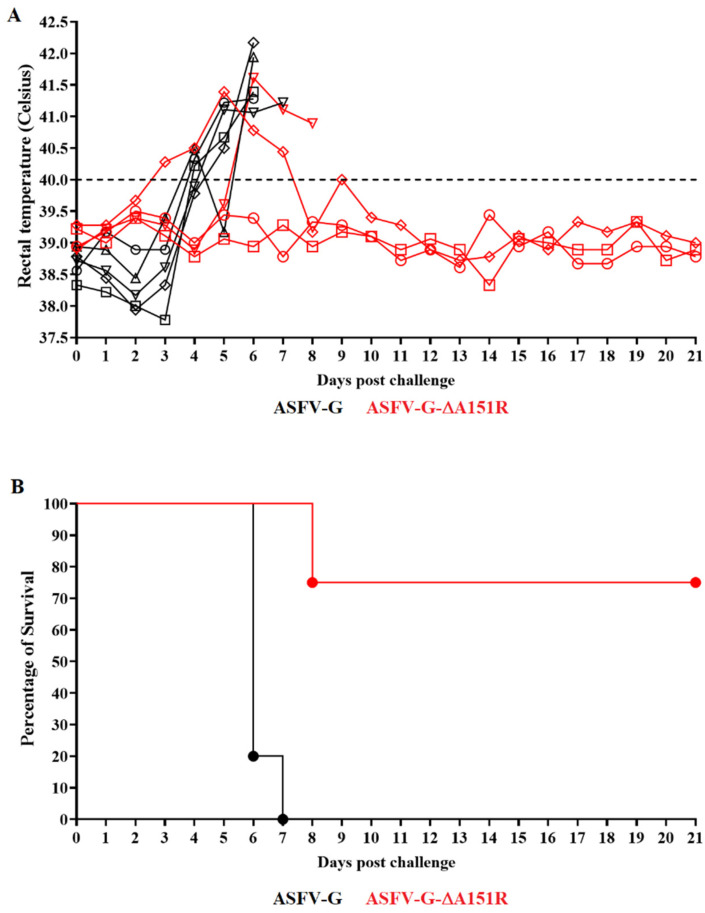
Evolution of body temperature (**A**) and mortality (**B**) in animals (4 animals/group) IM infected with 10^2^ HAD_50_ of ASFV-G-ΔA151R and challenged 28 days later with 10^2^ HAD_50_ of parental ASFV-G.

The onset of the disease in the control group occurred by days 4 to 5 post-challenge (dpc), and quickly progressed to a more severe clinical disease with all animals being euthanized by days 6 and 7 pc (Figure 8). Conversely, except for one animal that developed a clinical disease similar to that in the mock-vaccinated group, ASFV-G-ΔA151R-infected animals survived the challenge. Two of them remained clinically normal throughout the 21-day observational period. The third one presented a transient rise in body temperature between day 3 and 7 pc and remained asymptomatic after that until day 21 pc.

Viremia titers in mock-vaccinated and challenged animals followed the expected evolution along with the clinical signs. Titers were high (ranging between 10^7.3^ and 10^8.05^ HAD_50_/mL) at day 4 pi and remained high until animals were euthanized due to the severity of the clinical disease on day 7 pc (Figure 6). After the challenge, viremia titers of the ASFVG-ΔA151R-infected animals had a heterogenous presentation following the appearance of clinical signs of the disease. The animal that developed a lethal disease presented undetectable virus on day 4 pc and a sudden rise on day 7 pc (10^6.8^ HAD_50_/mL) before being euthanized the next day. The other three animals presented long viremias after the challenge and peaking titers by day 7 pc, which remained present at different levels until the end of the observational period (21 days pc) (Figure 6). Therefore, three out the four animals who survived ASFV-G-ΔA151R infection developed a protective immune response and survived the challenge with the virulent parental virus ASFV-G.

These results indicate that deletion of A151R from the genome of ASFV-G affects virus replication and virulence when experimentally inoculated in domestic swine. It is important to note that just a few genes (or group of genes) have been demonstrated to produce the attenuation of virulence when deleted from the genome of ASFV-G or its derivative viruses. The deletion of the 9GL gene (by itself or in combination with the UK gene deletion) [9,41], a six-gene deletion of the MGF360 and 530 members [7,11], the deletion of the I177L gene [6,42], the co-deletion of the CD2-like and UK genes [43], the combined deletion of genes L7L, L8, L9L, 304 L10L, and L11L [44], the deletion of QP509L/QP383R genes [45], and the individual deletions of genes A137R [12], I226R [38], A104R [39], E184L [46], and MGF110-9L [47] are the only reports describing attenuation of an ASFV-G strain. Therefore, A151R is one of the few genes experimentally demonstrated to be involved in the process of disease production by the ASFV Georgia isolate.

In summary, we determined that A155R is a non-essential gene, since its deletion from the ASFV-G genome did not significantly alter virus replication in cell cultures of swine macrophage cultures, the natural cell target during infection in swine infection. However, A155R is important for ASFV virulence in domestic pigs.

## Figures and Tables

**Table 1:** Swine survival and fever response following infection with ASFV-G-∆A151R and parental ASFV-G.

			Fever		
Virus (10^2^ HAD_50_)	No. of Survivors/Total	Mean Time to Death (± SD)	No. of Days to Onset (± SD)	Duration No. of Days (± SD)	Maximum Daily Temp, °C (± SD)
ASFV-G-∆A151R	4/5	21 *	16	5	40.94
ASFV-G	0/5	7 (0)	5.6 (0.55)	1.4 (0.55)	41.25 (038)

(*) Results for the only animal in the group that developed disease and needed to be euthanized on day 21 pi.

## Data Availability

Not applicable.
